# Eye tracking use in researching driver distraction: A scientometric and qualitative literature review approach

**DOI:** 10.16910/jemr.12.3.5

**Published:** 2019-09-30

**Authors:** Tina Cvahte Ojstersek, Darja Topolsek

**Affiliations:** University of Maribor, Faculty of Logistics, Celje, Slovenia

**Keywords:** eye tracking, driver behaviour, driver distraction, literature review, scientometrics

## Abstract

Many factors affect road safety, but research constantly shows that drivers are the major cause of critical situations that could potentially lead to a traffic accident in road traffic. Visual information is a crucial part of input information into the driving process; therefore, distractions of overt visual attention can potentially have a large impact on driving safety. Modern eye tracking technology enables researchers to gain precise insight into the direction and movement of a driver’s gaze during various distractions. As this is an evolving and currently very relevant field of road safety research, the present paper sets out to analyse the current state of the research field and the most relevant publications that use eye tracking for research of distractions to a driver’s visual attention. With the use of scientometrics and a qualitative review of the 139 identified publications that fit the inclusion criteria, the results revealed a currently expanding research field. The narrow research field is interdisciplinary in its core, as evidenced by the dispersion of publication sources and research variables. The main research gaps identified were performing research in real conditions, including a wider array of distractions, a larger number of participants, and increasing interdisciplinarity of the field with more author cooperation outside of their primary co-authorship networks.

## Introduction

Road accidents continue to be one of the major causes of deaths worldwide. In 2016, road injuries were the eighth top cause of deaths, killing 1.35 million people worldwide [1]. Many factors affect road safety, but research constantly shows that drivers are the major cause of critical situations that could potentially lead to a traffic accident in road traffic. For example, Singh [2] found that the driver is the cause of critical situations in 94 % of cases, 2 % can be attributed to vehicles, 2 % to the environment, while the remaining 2 % of critical situations are unexplained. These critical situations often lead to traffic accidents, where Dingus et al. [3] examined daily driving and traffic accidents that occurred during tracking of participants’ driving habits and found that almost 90 % of the causes of accidents can be attributed to driver-related factors. Given the prevalence of driver caused critical situations and road accidents, research into the process of driving and potential distractions to the process are imperative for enabling better driving safety.

Rupp [4] notes that driving is a complex activity consisting of many tasks of physical movements, psychomotor tasks, and sensory tasks that require the processing of appropriate visual and auditory information and cognitive and physical processes. As Lee, Young and Regan [5] note, driver distraction can be seen as any diversion of attention towards a non-driving related activity that diverts the driver away from the activities that are crucial for safe driving. Due to driving being an activity that demands attention and a series of reactions which are interconnected, every distraction that influences the driver can cause a disturbance in the whole driving process [6], which in turn can lead to critical situations or even traffic accidents. Singh [2] finds that 41 % of critical situations, caused by humans, can be traced back to errors of perception (inattention, distractions inside or outside of the vehicle, insufficient monitoring of the traffic environment and events). The attention of the driver, directed towards the traffic environment, traffic events, and the vehicle itself, is, therefore, one of the crucial aspects of traffic safety, since only sufficient driver attention ensures that the driver is getting all relevant information they need for making driving-related decisions. In a research of traffic accidents which occurred during everyday driving, Dingus et al. [3] found that the drivers were directing their attention towards distractions inside or outside of their vehicle in the six seconds leading up to an accident in as much as 68.3 % of recorded accidents, with the largest distraction being distractions that warrant the driver to redirect their visual attention away from the traffic environment and events, such as mobile phone use or searching for an object inside the vehicle. 

Visual attention is therefore of key importance for successful and safe driving. As early as the 70s of the 20th century, researchers estimated that as many as 90% of the information needed for driving is obtained through the visual channel [7]. Precise quantification of the amount of information that drivers acquire with vision in relation to other senses is practically impossible, since in the process of driving, in addition to visual information, drivers also obtain and process audio and kinaesthetic information, while at the same time interactions between all information and their processing are carried out at the cognitive level of the driver [8]. Additionally, drivers obtain visual information not only from their direct location of gaze (overt visual attention), but also covertly using their peripheral vision [9]. Research by Fort et al [10].that studied brain activity while driving has undoubtedly shown that when changes in the traffic environment and traffic events occur, the parts of the brain responsible for visual perception and processing are activated first, and that, based on these processes, other centres in the brain are activated, for example those for motor skills, coordination, and attention [11].

Research on distractions affecting the driver while driving is likely as old as the act of driving itself. The first researches of advertising boards, one of the most researched influences on visual attention drivers even today, appeared in the 1950s. Their findings were different and often contradictory, for example, Staffeld [12] and Rusch [13] found that there were significantly more traffic accidents on road sections and intersections with many billboards than in areas without them, while McMonagle [14] and Lauer and McMonagle [15] found that roadside advertising has no significant impact on the number of traffic accidents or on the act of driving. Several years later, research into drivers’ visual distractions began to appear. King and Sutro [16] were among the first and they concluded that the cause of one out of eight traffic accidents lies in a direct hindrance to the driver’s visual field, and cases where the driver turns his attention away from the traffic environment and events due to various distractions should also be added to this statistic. The first studies using eye tracking technology to explore the overt visual attention of drivers appeared in the middle of the 20th century, when gaze tracking was based on simpler cameras installed on the dashboard. The first studies go back as far as the 1970’s, when Mourant and Rockwell [17, 18] explored search and scan patterns of drivers with a head-mounted system with an eye-marker camera and a stabilization unit [19]. Graf and Krebs [20] used a different apparatus, consisting of an oculometer that superimposed the fixation locations on a video of the driving scene to research driver responded to headlamps. A while later, research using eye tracking technology in the field of traffic safety and specifically in the field of visual attention of drivers began to focus on the distractions that drivers encounter inside and outside of the vehicle. Crundall and Underwood [21] studied the differences in gaze direction, fixation duration, and horizontal and vertical spread of search, among experienced and beginner drivers under conditions of different cognitive loads as caused by variable road conditions. In one of the first large-scale driver distraction studies, Sodhi et al. [22] and Sodhi, Reimer, and Llamazares [23] studied the influence of frequent distractions on gaze direction and patterns of gaze movements with basic eye tracking equipment, composed of three cameras in the vehicle, one of which was mounted on the driver’s head. The main disadvantage of most of the early studies is the fact that due to the limitations of the equipment used, the analysis covered only data on gaze shifts inside a coordinate system without considering what the driver actually sees by applying the gaze data onto a video of the driver’s visual field. Modern eye tracking technology has largely surpassed these limitations and can consequently give researches important and accurate insights into the visual attention of drivers. 

In recent years, the use of eye tracking technology in the study of driver behaviour has been growing and expanding, as the ease of using this technology, its accuracy, and the quality and usability of the acquired data have vastly improved. It seems that the opportunities for eye tracking use in researching driver distractions are endless. For this reason, the purpose of this paper is to collect and analyse the studies carried out so far, which specifically examined distractions of drivers’ visual attention using eye tracking equipment. The literature review will focus on the methodological aspects of included scientific contributions to determine the ways in which eye tracking had been used in relevant research up to now. Moreover, we will analyse this narrow field of scientific research using basic scientometric procedures to determine the authors and topics that are prevalent in the field. Based on this, a summary of the main findings about the state of the research field will be given with suggestions for future use of eye tracking in the research of drivers’ visual attention and distractions will be presented. 

## Methods

When looking for previous scientific publications using eye tracking to analyse distractions that affect the driver while driving, Scopus and Web of Science databases were used. The search was performed in July 2019. These two databases were selected because they are the most commonly used, but at the same time differ in their coverage [24]. Therefore, using both instead of just one will broaden the scope of our preliminary search and represent a set of scientific publications covering the most commonly used criteria for demonstrating the scientific impact of publications. In both databases, we searched for publications with the search string (("eye track*" OR "eye movement*") AND driv* AND distract*) with no other restrictions. Since the main goal of this paper is to analyse scientific studies of driver distractions that used eye tracking as their main research tool, some exclusion criteria were set to ensure the inclusion of only relevant studies. Excluded were publications that:

- present only literature reviews;
- focus on theoretical aspects of using eye tracking, and conceptual papers;
- focus on the technical aspects of eye tracking technology or present patents;
- suggest the use of eye tracking technology, but do not demonstrate direct use of the technology in research;
- study modalities other than car road transport, and papers devoted to autonomous vehicle use;
- use eye tracking technology for research into drivers’ visual behaviour during driving without specifically studying distracting factors, e.g. development of a model for predicting distracted driving or driver drowsiness or researching gaze patterns of drivers in general;
- do not deal directly with examining driver distraction, but rather use eye tracking to examine design features of various elements in the traffic environment or vehicle, e. g. the design of traffic signs, changes in road geometry, user interface design of in-vehicle systems…

In Web of Science, a search was performed using the search string in the Topic field, which includes the title, keywords, and abstract. The search returned 372 results. In Scopus, the search, which included paper titles, keywords, and abstracts, returned 429 results. After deleting duplicates, 572 publications remained. The publications were reviewed, researchers read the abstracts and, where necessary, the methodological part. All publications were independently reviewed by two researchers and the publications that both excluded were taken out of the literature pool immediately, while the publications that the researchers didn’t agree on were reread and debated upon. The literature pool included 103 publications after this step. In order to ensure inclusion of all relevant scientific publications, reference lists of the 30 top cited publications from the current literature pool were also reviewed to find more relevant scientific publications that were not identified based on Scopus and WOS search. Through this step, additional 36 publications were added into the literature pool. Taking into account the exclusion criteria, 139 scientific publications that present research of driver distraction with the use of eye tracking technology were selected to be included in the analysis. 

**Figure 1:**
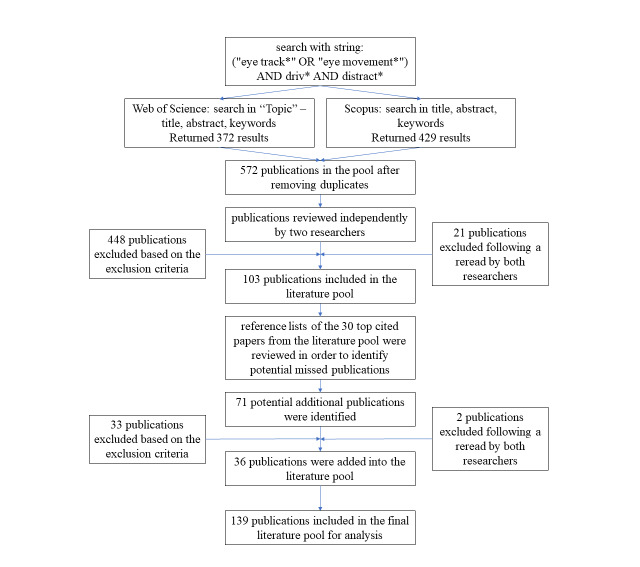
Publication selection process.

The process of selecting the included studies is shown in Figure 1. 

The literature analysis was performed in two steps. First, we focused on bibliometric analysis showing basic parameters such as publication year and source journals, and science mapping, which utilized the VOSviewer tool [25] Science mapping is a procedure used to analyse and visualize a specific field of scientific research [26] Due to software limitations, input data for science mapping was exported from Scopus only, which resulted in an omission of three publications that are not indexed in Scopus, and consequently, all analysis in VOSviewer was performed on a literature pool of 136 publications Several analyses were performed using VOSviewer: citation analysis, co-citation analysis, bibliographic coupling analysis, co-authorship analysis, and co-occurrence analysis of keywords.

Furthermore, the analysis focused on how the research presented in the papers used eye tracking technology to research driver distraction and what variables were most often used. Therefore, we gathered information about the observed distractions, independent and dependent research variables, type of eye tracking used, and whether the research was performed in a driving simulator or on real roads. Based on this, an analysis of the key elements of the included publications was performed and presented. Additionally, Appendix A shows the whole literature pool and the observed factors of eye tracking use.

## Results

### Bibliographic analysis and science mapping 

**Figure 2:**
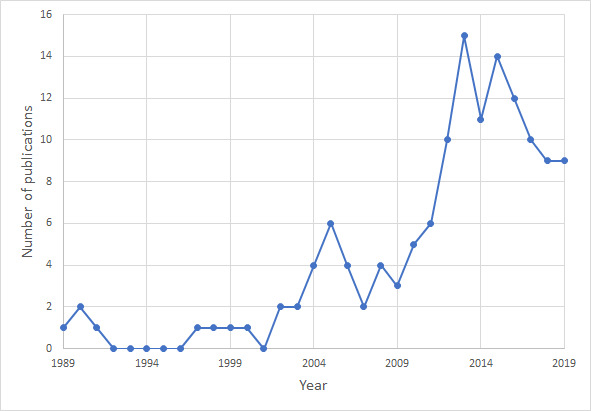
Number of publications per year.

Even though eye tracking tools have existed for quite some time, their extensive use in researching driver distractions began rising after the year 2002 when yearly output on the topic of driver distraction research using ET began rising. Before that, only 9 publications were published. From 2011 on, a significant increase in output on the field can be seen with publishing activity reaching its peak in 2013. The overall trend of a growing number of publications is seen, as shown in Figure 2. The data for 2019 is not yet complete, since the data for the present paper was collected in the middle of the year. 

Out of the 139 included publications, 33 were published in conference proceedings and 106 in 10 different scientific journals. The total number of unique sources of publications is 50. A detailed publication number for the most productive sources that published 3 or more publications from the literature pool is shown in Table 1.

**Table 1:** Sources.

Source title	Number of papers
Transportation Research Part F: Traffic Psychology and Behaviour	23
Accident Analysis and Prevention	21
Applied Ergonomics	14
Proceedings of the Human Factors and Ergonomics Society	8
Transportation Research Record	8
Human Factors	7
AutomotiveUI: International Conference on Automotive User Interfaces and Interactive Vehicular Applications	4
Journal of Experimental Psychology: Applied	4
Ergonomics	3

To identify papers with the largest impact in the field, citations in the Scopus database were also examined. 14 publications have more than 100 citations with Strayer, Drews, & Johnston [27] being the most cited with 608 citations. The top ten cited publications are shown in Table 2.

**Table 2:** Most cited papers.

Authors and publication year	Number of citations
Strayer, Drews, & Johnston, 2003 [27]	608
Engström, Johansson, & Östlund, 2005 [28]	392
Brookhuis, de Vries, & de Waard, 1991 [29]	363
Recarte & Nunes, 2003 [30]	357
Recarte & Nunes, 2000 [31]	282
Victor, Harbluk, & Engström, 2005 [32]	275
Lamble, Kauranen, Laakso, & Summala, 1999 [33]	265
Harbluk, Noy, Trbovich, & Eizenman, 2007 [34]	244
Horrey, Wickens, & Consalus, 2006 [35]	201
Wikman, Nieminen, & Summala, 1998 [36]	145

**Figure 3:**
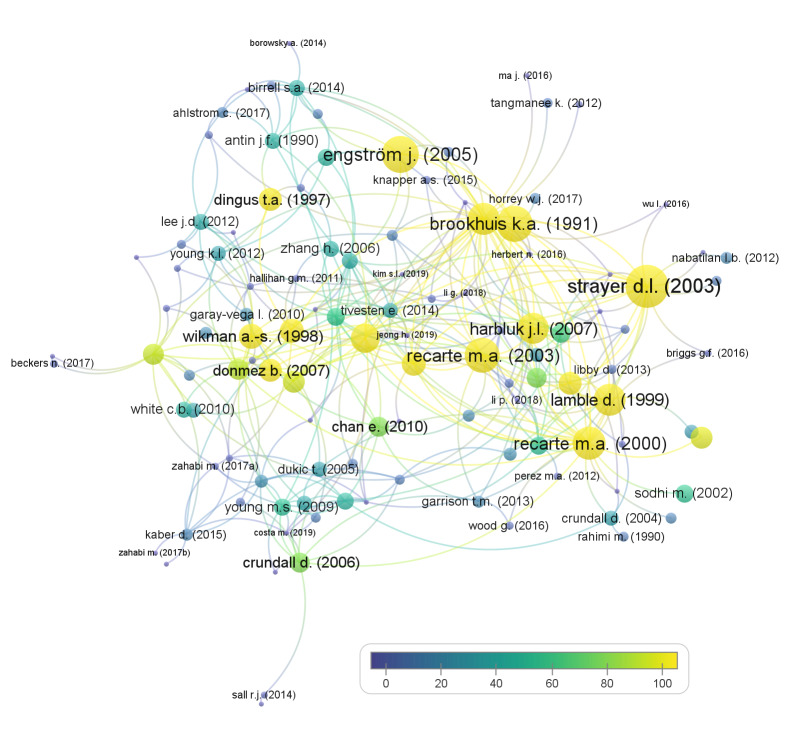
Citation mapping.

Citation mapping was performed using the VOSviewer tool and is based on citations of 136 papers in the literature pool in the Scopus database. Each of the 136 publications is represented with a node (circle) and named with the first author’s surname and publication year on Figure 3 The size of the node and its colour show the number of citations of each paper, and the lines between nodes show a citing connection between two papers The largest cluster of citations that emerged in the literature pool contains 115 items, as seen in Figure 3

**Figure 4:**
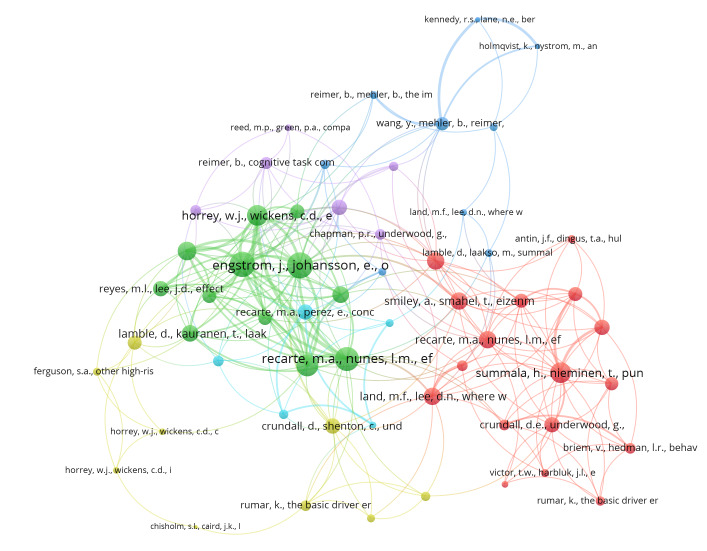
Co-citation analysis.

**Figure 5:**
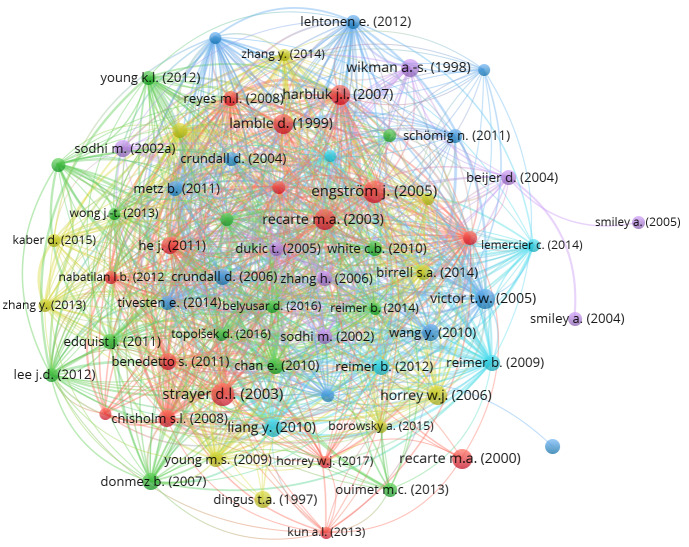
Bibliographic coupling.

According to van Raan [37], citation analysis can be performed on the level of the secondary network as well, where we can identify co-citations (where two publications are cited together by another publication) and bibliographic coupling (where two publications have references in common). These two secondary network analyses were performed in VOSviewer and the results are shown in Figure 4 and Figure 5. 

Co-citation analysis analyses how often two publications are cited together in another publication. 4057 publications that were cited by publications in the literature pool of the present research were first identified (for example, Engström, Johansson, and Östlund [28] and Strayer, Drews, and Johnston [27] are both cited by Edquist, Horberry, Hosking, and Johnston [38]). Only publications that were cited at least three times were included in the co-citation analysis, meaning 58 items. The co-citation network is shown in Figure 4. Similar colours of the nodes represent papers that are connected, meaning they are cited together in other publications more often and therefore likely similar in topic and relevant for the research field, and the link weight presents the co-citation strength. The network is formed out of six clusters of papers, often co-cited.

Bibliographic coupling analysis was performed on the literature pool by including only publications that have at least 10 citations in the Scopus database. The largest set of interconnected publications includes 65 items, as shown in Figure 5

Each node represents an included paper and each line represents a bibliographic coupling occurrence, meaning that the connected papers share a common reference (for example, both Dukic, Ahlstrom, Patten, Kettwich, & Kircher [39] and Edquist, Horberry, Hosking, and Johnston [38] reference Crundall, Van Loon, and Underwood [40]). The size of each node presents its relative number of citations, and the weight of a link (line) presents the relative amount of references the two connected papers share. From the analysis in VOSviewer, it is evident that six clusters of publications emerged, presenting papers that are connected and have more references in common, which points to their relative similarity in topics. These are represented in the graphic with the same colour. A graphical representation of the bibliographic coupling relations in the literature pool is shown in Figure 5.

**Figure 6:**
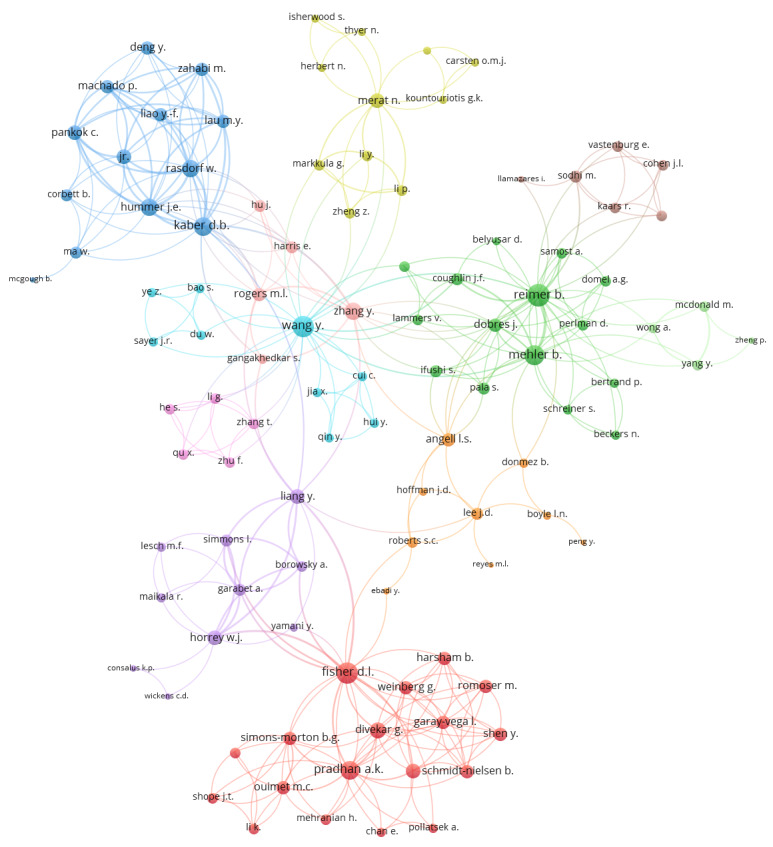
Largest cluster of co-authorship.

Altogether, 360 authors were identified as contributing to the pool of research papers after manual consolidation of results due to different issues with author names (e.g. engström j. and engström j.a. were consolidated into engström j.a.. The most productive authors up to now are Reimer B. with 11 publications, Kaber D. D., Mehler B., and Fisher D. L. with eight publications each, Liang Y. with seven publications and Horrey W. J. and Wang Y. with six publications each. If all contributing authors are included in the analysis, the largest cluster of co-authorship with 104 authors that emerges is shown in Figure 6. VOSviewer produced a co-authorship scheme as shown in Figure 6. Each node represents an author, and the larger the size, the larger the number of contributions into the literature pool. The line weight presents the amount of publications the authors have co-authored.

VOSviewer identified 1077 keywords, which included the author and index keywords. Keywords were looked through and synonyms were joined before the visualization process was run (e.g. car drivers and automobile drivers were joined into car drivers, eye tracking and eye-tracking were joined into eye tracking), as well as keywords with a common meaning (e.g. driving simulation, driving simulator, driving simulators, and driving simulator study were joined into driving simulation). This reduced the number of keywords to 951.

**Figure 7:**
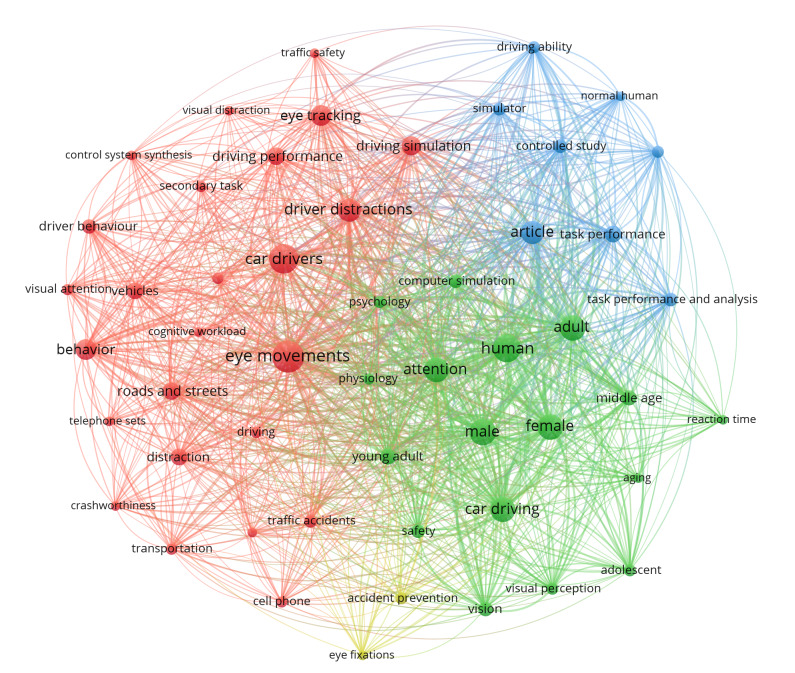
Co-occurrence of index and author keywords.

The keywords with most occurrences were “eye movements” with 78 occurrences, “car drivers” with 68, and “human” with 62. All keywords that were used at least ten times (52 keywords) are shown in Figure 7, where each circle presents a detected keyword, the size of the node points to the number of occurrences of the keyword, and the links show which keywords appear together in publications. We can identify four clusters of keywords, meaning that these keywords have common themes.

**Figure 8:**
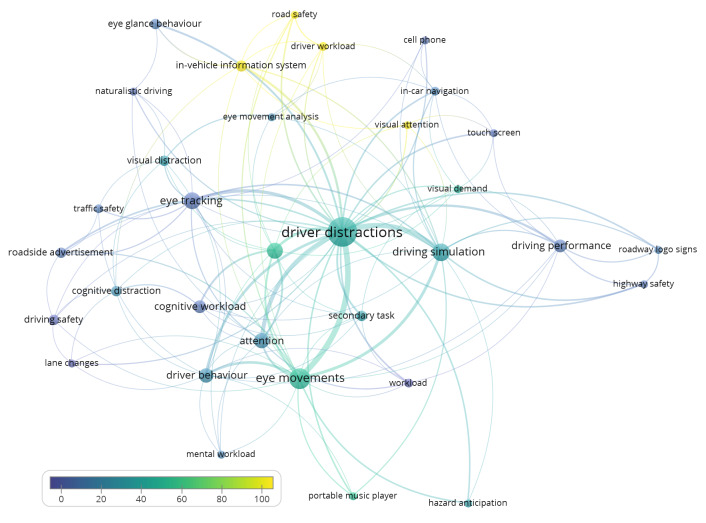
Co-occurrence and citation count of author keywords.

Author keywords are a subsection of all keywords that VOSviewer identified, these are the keywords identified by the authors as representative of the papers’ contents. VOSviewer identified 288 unique keywords in the literature pool. Once again, synonyms and keywords with a common meaning were identified and merged, which left 253 keywords for analysis input. If we set the minimum number of a keyword occurrence to three, 33 author keywords are included in the analysis, which is shown in Figure 8. The three most used author keywords are “driver distractions”, “eye movements” and “driving simulation”. Once again, the circles show keywords and the size of a frame points to its relative number of occurrences. The colour of a circle shows how many times papers with that keyword have been cited up to now, with yellow being the most cited keywords and blue the least cited. Even though “driver workload” and “visual attention” are among the least used keywords, they are associated with the most cited papers, and on the other hand, “driver distractions” and “eye tracking” are associated with the most papers but are not among the most used in association with citations of papers.

### Use of eye tracking in driver distraction research 

A table that shows Authors, publication year, source, observed distractions, observed independent and dependent variables, number of participants, setting, and type of eye tracking used, can be found in Appendix A. For shortening purposes, all elements of the table in the appendix are presented with codes which will be give alongside the results in this chapter.

In the analysed publications, some used driving simulators, some on-road setting, some both, and some utilized a simpler approach by showing pre-recorded video clips of driving situations to participants. Out of the analysed publications, research was performed in a real road setting in 44 publications, 81 publications utilized a driving simulator of various sophistication, four publications used both simulators and on-road settings, and nine publications used only video recordings. Out of the 134 papers that had information about the number of participants’ data included in the eye tracking analysis available, the range of participants is from one to 123. The mean number of participants is 28.96 with a standard deviation of 21.58. If we only look at research that was done in a driving simulator, the mean number of participants is 27.84 with a standard deviation of 17.02 (min 5, max 120), and research performed on real roads had a mean of 26.05 participants (sd 23.63, min 1, max 123). Based on these statistics and taking into account the large standard deviations, we can conclude that the number of participants varies largely and that the research setting does not seem to have a direct influence on the number of included participants. 

As for the eye tracking technology used, we grouped the used technology into two broad categories: head-mounted, wearable eye tracking systems, usually in the form of glasses or sometimes implemented with a use of a helmet, and remote eye tracking systems of various configurations, ranging in sophistication from a basic camera that records eye movements and where eye tracking data has to be coded manually, to multi-camera dashboard set ups that automatically code eye movements onto a recording of the driver’s visual field. Out of the 139 included publications, 53 publications report results, obtained with wearable eye tracking systems (coded as HMGL in the Appendix), 80 with remote or dashboard mounted systems (RDSB), 1 used both systems, and information is not available for 5 publications. 

Since driver distraction research is at the core of the present research, the next step focused on examining, what types of driver distraction publications in the literature pool focused on. The analysis is based on grouping distractions with common elements or influences on the driver. The most common distractions, such as cell phone use for texting, were categorized separately, and other, less often used distractions or generic tasks such as the n-back task, were grouped together. Table 3 presents the results of driver distraction analysis along with the number of publications which focus on a distraction or group. Cognitive distractions are by far the most researched, where researchers use generic cognitive tasks to induce a high cognitive load. As for specific elements, various roadside advertisements and information signs are the most commonly observed distraction outside of the vehicle, and cell phone use inside the vehicle. 

**Table 3:** Frequency of observed driver distractions.

Code in Appendix A	Observed driver distractions	Frequency
OTCOG	other cognitive tasks (minimal manual or visual effort), e.g. n-back task, sound counting	44
ADSG	roadside advertising signs, billboards, information or logo signs, regardless of type (static, electronic, video...)	23
OTVIS	other visual tasks (minimal cognitive or manual effort), e.g. target search	18
PHCNV_h	hand-held cell phone conversation	17
PHDIA_h	hand-held cell phone dialling task	15
IVIS	in-vehicle information system or driver support systems, e.g. driving support and information, various control features	15
NAV_iv	in-vehicle navigation system	14
RADMS	radio or in-vehicle built in music system use, e.g. radio tuning, CD change, music search	14
OTTE	other elements of the traffic environment, e.g. pedestrians	14
PHCNV_hf	cell phone conversation using a hands-free system	9
NAV_ptb	portable navigation system	8
PHOTH	other cell phone/smartphone tasks, e.g. browsing social networks, app use	7
IVPAR	adjusting or reading various in-vehicle parameters, such as climate control, lights, speed control	6
PMP	portable music player	5
PHTXT	sending and receiving SMS messages via cell phone (texting)	5
OTMAN	other manual tasks, e.g. in-vehicle search tasks	5
PHDIA_hf	cell phone dialling task using a hands-free system	4
OTIV	other elements inside the vehicle	4
PASS	passenger	3

The observed distractions were studied with various research procedures and analysis models. By analysing the variables that were the research focus, we can gain a better understanding of the procedures used in the field of driver distraction research using eye tracking. First, Table 4 presents the frequency of various independent variables that were used for analysis of driver eye movements. All publications compare the baseline driving condition (without distractions) to the experiment condition as a basic research procedure, that is why the baseline vs. experimental condition variable is not shown in the table below. Besides that, the type of task, target, or distraction, is the most commonly used independent variable, which gives researchers an opportunity to compare various influences to driver visual attention based on the content or configuration of the distraction. 

**Table 4:** Frequency of used independent variables.

Code in Appendix A	Independent variable	Frequency
TYPT	type of task/target/distraction (e.g. type of sign, contents of advertisements, specific instructions for the task, type of feedback).	95
ROAD	type of road or road geometry	30
TSKD	task difficulty (various difficulties of the same task)	28
MODT	modality of technology (e.g. type of navigation system, handheld or hands-free operation, touch or speech control)	26
HZRD	type of hazard / critical event (e.g. pedestrian on road, lead vehicle braking)	24
TRCON	traffic conditions, e.g. density, configuration, weather	16
AGE	age of participant	15
LOCOV	location of target outside vehicle	14
OTHR	other	12
DREXP	driving experience of participant	11
OTPAR	other participant variables (e.g. familiarity with the distraction, attitude towards risk)	11
GEND	gender	9
LOCIV	location of target inside vehicle	7
TIME	time of day	7

Table 5 presents the core of research in the field, observed variables that describe and analyse driver eye movements. The analysis of these variables showed that the basic unit of analysis varies among publications, since some use glances and some fixations as their primary focus. According to the ISO 15007-1:2014 standard, fixations and glances are not the same [41]. This standard (and the ISO/DIS 15007 that is planned to replace the 2014 standard) defines a fixation as a short static alignment of the eyes to a particular point, and a glance as a time section where the gaze is maintained inside a selected area of interest (meaning that a glance can consist of more fixations and saccades). Glances, their frequency, duration, and pattern, are used most often. It is worth noting however that there is a possibility that not all included publications used the same differentiation between glances and fixations.

**Table 5:** Frequency of used observed eye movement related variables.

Code in Appendix A	Eye movement related observed variables	Frequency
GL_num	number (frequency) of glances to a specific target or area of interest in a set time period	49
GL_adur	average duration of glances to a specific target or area of interest in a set time period	44
GL_patt	glance or fixation patterns or heat map	31
FIX_num	number (frequency) of fixations on a specific target or area of interest in a set time period	27
VAR_hor	variance of horizontal range of fixations	26
GL_%	percentage of glances to a specific target or area of interest in a set time period	25
FIX_adur	average duration of fixations on a specific target or area of interest in a set time period	24
GL_tot	total time spent glancing on a target in a set time period	23
GL_%t	percentage of time glancing to a specific target or area of interest in a set time period	21
GL_over	number or percentage of glances over a set threshold (most often 2 seconds)	19
VAR_vert	variance of vertical range of fixations	19
GL_max	longest duration of a glance to a specific target or area of interest in a set time period	18
OTHR	other measures	17
SACC	saccade measures - duration, speed, amplitude...	17
FIX_tot	total time spent fixating on a target in a set time period	14
GZ_andev	deviation of gaze angle from a set point (most often the center of the road)	13
PUP_size	average pupil size (diameter) in a set time period	13
FIX_%	percentage of fixations on a specific target or area of interest in a set time period	12
FIX_%t	percentage of time fixating to a specific target or area of interest in a set time period	10
BL_rate	blink rate in a set time period	10
TTFF	time to first fixation to the target or area of interest	7
BL_dur	blink duration	5
FIX_max	longest duration of a fixation to a specific target or area of interest in a set time period	4
FIX_over	number or percentage of fixations over a set threshold (most often 2 seconds)	1

Other observed variables not directly connected to eye movements, and their frequencies, are shown in Table 6. Measures of the vehicle’s lateral lane position and speed that point to the drivers’ driving performance, and measures of drivers’ performance on the secondary task (distraction) are commonly used in combination with eye tracking measures in order to further research the influence of distraction on driver performance

**Table 6:** Frequency of used other observed variables.

Code in Appendix A	Other dependent variables	Frequency
LATP	lateral position measures (e.g. lane exceedances, variability of lateral lane position, time out of lane)	60
TSK_perf	secondary task performance (e.g. completion rate, number or correct responses)	58
SPE_stat	vehicle speed measures of a stationery kind (e.g. average speed, deviations of average speed)	51
WRKL_s	subjective workload ratings	31
RET	reaction time to presented task/hazard appearance	31
STWH	steering measures (e.g. steering wheel reversal rate, steering entropy)	30
TSK_t	task completion time	28
OTHR	other	24
HWTC	headway distance or time to collision	20
SPE_ch	vehicle speed measures that point to speed changing (e.g. acceleration events, braking force)	18
INC	incident measures (e.g. critical errors, traffic rules violations)	14
HRT	heart rate measures	8
PERF_s	subjective performance rating	6
SKC	skin conductance measures	6

## Discussion

Distracted driving is undoubtedly a rising issue that needs to be tackled by scientists and practitioners worldwide. A deeper understanding of the effect of distractions on the drivers’ visual attention is made possible by using eye tracking technology, which has become portable and highly accurate in the last decade and therefore enables research that was almost impossible not so long ago.

To present the current state of the field of eye tracking use in researching distractions to drivers’ visual attention, this paper focused on science mapping and bibliometrics to describe the field and find its main characteristics. Its main conclusions can be summed up as follows:

- Generally speaking, eye tracking is currently being used as a supportive technology in driver distraction research. Given its potential for an accurate insight into the overt visual attention of drivers, it can be expected that this field of research is expanding and will likely continue to grow in the next years. Currently, 139 papers that focus on examining the influence of distractions on drivers’ visual attention can be found in WOS and Scopus, and the overall publication trend is on the rise.
- The field is interdisciplinary in its core, which is also reflected in the source publications where papers are being published. Journals and conferences that publish papers from the field are predominantly focused on psychology and human factors, transportation, safety, and ergonomics.
- Citation analysis shows that by far the most referenced paper is on the topic of cell phone distractions, which are also the focus of three other publications among the top ten cited. An analysis of citing among papers in the literature pool shows that the field is relatively interconnected since 115 out of the 136 included publications share at least one citation link. This is not surprising given the narrow focus of the present analysis. Secondary citation network analysis (co-citation and bibliographic coupling) further proves the above finding and additionally forms six clusters of connected publications which are most often cited together or share common references.
- The amount of cooperation among authors in the field, especially outside of their primary co-authorship network, is relatively low as shown by the co-authorship analysis. The co-authorship scheme shows that some co-authorship clusters have formed but are interconnected only with occasional collaborations.
- Four keyword clusters can be identified. One presents research, focused on utilizing driving simulators; the second seems to be focused on evaluating visual attention while taking into account participant factors such as age, gender, and psychological factors; the third, smallest cluster, focuses on accident prevention; and the fourth, largest, focuses on safety and specific distractions and tasks. As expected, “eye movements”, “driver distractions”, “car drivers” and alike are the most commonly used keywords, and these are also keywords used in the most cited papers from the field as shown by the analysis of author keywords.
- A little under a third of the included papers present research, performed in real road conditions, other papers utilize driving simulators of various sophistication levels, prerecorded video recordings of driving situations, and four publications utilize both simulators and on-road research. On average, research includes roughly 29 participants, with the average number of participants being slightly higher for research in driving simulators (27.84 versus 26.05 for research in real conditions), but given the large standard deviations, it does not seem that research settings influence the number of included participants.
- An overall analysis of the research variables points to the fact that cognitive distractions are most researched, followed by visual ones. Cell phones and various IVIS systems are at the centre of in-vehicle distraction research while advertisements and information signs dominate research outside the vehicle. Most papers only include one distraction or task type. In addition to eye tracking parameters, the effects of driver distraction are often analysed by using complementary variables, such as parameters of driving performance and task performance. Another point worth mentioning is the use of glances or fixations as the basic eye movement parameter. There seems to be a lack of consensus on the field on which unit of measurement to use. We did notice however that a lot of research lately uses the definition of glances and fixations as was put forward in the ISO standard on measuring driver visual behaviour ( 41 ), where fixations are seen as a static point of gaze focus and glances as a set of fixations and saccades inside a predefined area of interest.

Overall, this paper has shown that the field of eye tracking use in driver distraction research is an emerging field with space for improvement and collaboration. The included publications present the main publications in the field and the authors took every possible step to include as much relevant sources as possible, but a possibility exists that some relevant sources have nevertheless been omitted. We estimate however that the coverage of the resent literature review is sufficient to give meaningful insight into the state of the art on the topic of driver distraction research using eye tracking. 

 Future research recommendations that would fill the gaps as identified in the paper include performing research in real conditions, including a wider array of distractions and comparing them to one another, a larger number of participants, and increasing interdisciplinarity of the field with more author cooperation outside of their primary co-authorship networks. As new potential distractors emerge every day, we conclude that eye tracking is a good tool to evaluate the effects these distractors have on a driver and especially on his overt visual distraction.

### Ethics and Conflict of Interest

The authors declare that the contents of the article are in agreement with the ethics described in http://biblio.unibe.ch/portale/elibrary/BOP/jemr/ethics.html and that there is no conflict of interest regarding the publication of this paper. 

## APPENDIX A: In-depth analysis of papers in the literature pool

**Table:**

Authors, publication year	Observed driver distractions	Setting	Independent variables	Eye movement related observed variables	Other dependent variables	Number of participants, included in the eye movement analysis	ET type
Ahlstrom & Kircher, 2017 [42]	IVIS	on-road	TYPT	TTFF; GL_adur; GL_max; GL_%t; OTHR; GL_over	none	10	RDSB
Antin, Dingus, Hulse & Wierwille, 1990 [43]	NAV	on-road	MODT; GEND; DREXP; TRCON	GL_adur; GL_patt	TSK_t; TSK_perf; STWH; SPE_ch; LATP	32	RDSB
Aust, Dombrovskis, Kovaceva, Svanberg, & Ivarsson, 2013 [44]	PHDIA_h; NAV_iv; RADMS	on-road	LOCIV	GL_tot; GL_adur; GL_max	none	35	RDSB
Beckers, Schreiner, Bertrand, Mehler, & Reimer, 2017 [45]	NAV_iv; NAV_ptb	driving simulator	MODT; TYPT	GL_adur; GL_over; GL_tot	WRKL_s; TSK_t; LATP; SPE_stat; RET; TSK_perf; HRT; SKC	24	RDSB
Beijer, Smiley, & Eizenman, 2004 [46]	ADSG	on-road	TYPT; GEND; LOCOV; OTPAR	GL_adur; GL_max; GL_num; GZ_andev	none	25	HMGL
Belyusar, Reimer, Mehler, & Coughlin, 2016 [47]	ADSG	on-road	AGE; GEND; TYPT	GL_num; GL_over; GL_adur	SPE_ch; STWH; LATP	123	RDSB
Benedetto, Pedrotti, Minin, Baccino, Re, & Montanari, 2011 [48]	IVIS; OTVIS	driving simulator	TSKD; TYPT	BL_rate; BL_dur; PUP_size	WRKL_s; RET; TSK_perf	15	HMGL
Birrell & Fowkes, 2014 [49]	PHOTH	on-road	TYPT	GL_num; GL_%; GL_adur; GL_max; GL_%t; GL_over; GL_patt	none	15	RDSB
Blanco, Hankey, & Chestnut, 2005 [50]	RADMS; IVPAR; PHDIA_h; NAV_iv	both	TYPT	GL_num	LATP; SPE_ch; TSK_perf; TSK_t	89	RDSB
Borowsky, Horrey, Liang, Simmons, Garabet, & Fisher, 2014 [51]	OTVIS	driving simulator	TSKT; HZRD	GL_num	SPE_stat; WRKL_s; TSK_perf	56	HMGL
Borowsky, Horrey, Liang, Garabet, Simmons, & Fisher, 2015 [52]	OTCOG	driving simulator	TSKT; ; HZRD	GL_num	WRKL_s; PERF_s	12	HMGL
Borowsky, Horrey, Liang, Garabet, Simmons, & Fisher, 2016 [53]	OTCOG; OTVIS	driving simulator	TSKT; HZRD	GL_num; GL_adur	SPE_stat; WRKL_s; PERF_s	56 (14 per condition)	HMGL
Briggs, Hole, & Land, 2016 [54]	OTCOG	video clips	LOCOV	VAR_hor; VAR_vert; FIX_num	TSK_perf; RET; OTHR	46	HMGL
Brookhuis, de Vries, & de Waard, 1991 [29]	PHCNV_h; PHCNV_hf; OTCOG	on-road	ROAD; TRCON; MODT; AGE	GL_num	LATP; SPE_stat; STWH; HRT; WRKL_s; TSK_perf	12	RDSB
Chan, Pradhan, Pollatsek, Knodler, Fisher, 2010 [55]	OTTE; OTMAN; PHDIA_h	driving simulator	DREXP; TYPT	GL_adur; GL_max; GL_over; GL_tot	TSK_t; SPE_stat	24	HMGL
Chiang, Brooks, & Weir, 2004 [56]	NAV_iv	on-road	TSKD; ROAD	FIX_adur; FIX_%t; FIX_tot; FIX_num	TSK_t; TSK_perf; LATP; SPE_stat; WRKL_s	10	RDSB
Chisholm, Caird, & Lockhart, 2008 [57]	PMP	driving simulator	TSKD; TYPT; HZRD	GL_num; GL_adur	STWH; LATP; TSK_perf; RET; INC	19	HMGL
Chisholm, Caird, Lockhart, Teteris, & Smiley, 2006 [58]	RADMS; PHDIA_h; PHCNV_h; OTMAN	driving simulator	DREXP; HZRD	FIX_num; GL_tot; FIX_%t	RET; INC	40	HMGL
Colon, Rupp, & Mouloua, 2013 [59]	OTTE	driving simulator	TYPT; LOCOV	FIX_tot	STWH	54	RDSB
Costa, Bonetti, Vignali, Bichicchi, Lantieri, & Simone, 2019 [60]	ADSG	on-road	TYPT; ROAD; LOCOV	FIX_num; FIX_adur; OTHR	SPE_stat	15	HMGL
Crundall, Van Loon, & Underwood, 2006 [40]	ADSG	video clips	LOCOV; TYPT; HZRD	FIX_num; TTFF; FIX_tot; FIX_adur	OTHR; TSK_perf	32	HMGL
Crundall, Shenton, & Underwood, 2004 [61]	OTTE	driving simulator	TYPT; TIME; TRCON	FIX_adur; VAR_hor; VAR_vert; FIX_%t	SPE_stat; INC	15	HMGL
Desmet & Diependaele, 2019 [62]	PHCNV_hf	on-road	ROAD	FIX_num; FIX_adur; SACC; VAR_hor; VAR_vert	SPE_stat; LATP; HWTC	26	HMGL
Dingus, Hulse, Antin, & Wierwille, 1989 [63]	NAV_iv; IVPAR; RADMS; OTTE	on-road	TYPT; ROAD; TRCON; DREXP; AGE; GEND	GL_num; GL_adur; GL_tot	LATP; STWH; SPE_ch; TSK_t; TSK_perf	32	RDSB
Dingus, Hulse, Mollenhauer, Fleischman, Mcgehee, & Manakkal, 1997 [64]	NAV_iv	on-road	MODT; AGE; ROAD; TRCON; DREXP	GL_%t; GL_adur; GL_over	LATP; SPE_ch; TSK_perf; TSK_t; LATP; INC; STWH; WRKL_s	30	RDSB
Divekar, Pradhan, Pollatsek, & Fisher, 2012 [65]	ADSG	driving simulator	DREXP; HZRD; TYPT	GL_adur; GL_over; GL_num	LATP; SPE_ch	48	HMGL
Dong, Ma, Zhang, Zhang, & Wang, 2019 [66]	NAV_ptb; PHOTH	on-road	MODT; LOCIV	FIX_num; FIX_adur; GL_patt; BL_rate; BL_dur	SPE_ch; HWTC; LATP	20	HMGL
Donmez, Boyle, & Lee, 2007 [67]	IVIS	driving simulator	AGE; HZRD; TYPT	GL_num; GL_adur; GL_max	RET; HWTC; SPE_ch; STWH; SPE_stat; WRKL_s; PERF_s	29	RDSB
Dukic, Hanson, Holmqvist, & Wartenberg, 2005 [68]	OTIV		TYPT; LOCIV	GL_tot; GL_num	STWH; OTHR	8	HMGL
Dukic, Ahlstrom, Patten, Kettwich, & Kircher, 2013 [39]	ADSG	on-road	TIME; TYPT; LOCOV; OTHR	OTHR; FIX_num; FIX_max; FIX_adur	SPE_stat; LATP; HWTC	41	HMGL
Ebadi, Fisher, & Roberts, 2019 [69]	PHCNV_hf	driving simulator	HZRD; ROAD; TYPT	GL_num; OTHR	none	24	HMGL
Edquist, Horberry, Hosking, & Johnston, 2011 [38]	ADSG	driving simulator	TYPT; AGE; DREXP	FIX_%t	TSK_t; TSK_perf; WRKL	48	RDSB
Engström, Johansson, & Östlund, 2005 [28]	IVIS; OTCOG; OTVIS	both	TYPT; TSKD; ROAD	GZ_andev	TSK_perf; SPE_stat; LATP; STWH; SKC; HRT; PERF_s	48 in simulator, 24 on road	RDSB
Gable, Walker, Moses, & Chitloor, 2013 [70]	PMP	driving simulator	TYPT; OTPAR; DREXP	FIX_tot; GL_patt	LATP; TSK_t; TSK_perf; WRKL_s; PERF_s; OTHR	26	HMGL
Garay-Vega, Pradhan, Weinberg, Schmidt-Nielsen, Harsham, Shen, Divekar, Romoser, Knodler, & Fisher, 2010 [71]	RADMS	driving simulator	MODT; TYPT	GL_tot; GL_num; GL_over; OTHR	TSK_t; TSK_perf; LATP; WRKL_s	17	RDSB
Garrison & Williams, 2013 [72]	PHCNV_hf	driving simulator	TYPT	GZ_num; GZ_adur	SPE_stat; SPE_ch; LATP; STWH; TSK_perf	20	RDSB
Harbluk, Noy, Trbovich, & Eizenman, 2007 [34]	OTCOG	on-road	TSKD	GL_num; GL_%t	WRKL; SPE_ch; OTHR	21	HMGL
Hallihan, Mayer, Caird, & Milloy, 2011 [73]	IVIS	driving simulator	OTHR; MODT	GL_num; GL_adur; GL_%t; GL_over	SPE_ch; OTHR	14	HMGL
Hashash, Abou Zeid, & Moacdieh, 2019 [74]	PHOTH; TXT	driving simulator	HZRD; TYPT	FIX_num; FIX_adur; FIX%t; GL_patt	SPE_stat; LATP; RET; WRKL_s; PERF_s	26	RDSB
He, Becic, Lee, & McCarley, 2011 [75]	OTCOG	driving simulator	OTPAR; TRCON	VAR_hor; VAR_vert	OTHR; LATP; SPE_stat; HWTC	18	RDSB
Herbert, Thyer, Isherwood, & Merat, 2016 [76]	OTCOG	driving simulator	TYPT; TSKD	GZ_andev; FIX_%	SPE_stat; HWTC; LATP; STWH; TSK_perf	36	RDSB
Horrey, Lesch, Garabet, Simmons, & Maikala, 2017 [77]	OTCOG	driving simulator	TYPT; HZRD	PUP_size	HWTC; LATP; SPE_stat; RET; TSK_perf; HRT; OTHR	31	HMGL
Horrey, Wickens, & Consalus, 2006 [35]	PHCNV_hf; PHDIA_hf	driving simulator	TSKD; TRCON	FIX_%t	LATP; RET	8	RDSB
Hudák & Madleňák, 2017 [78]	ADSG	on-road	LOCOV; TYPT	GL_adur; GL_%t; OTHR	OTHR	3	HMGL
Jeong., Kim, Yu, Suh, Kim, & Suh, 2013 [79]	NAV_ptb	driving simulator	MODT; LOCIV	GL_patt	SKC; OTHR	15	RDSB
Jeong & Liu, 2019 [80]	OTCOG; OTVIS	driving simulator	ROAD; TYPT; TSKD	GL_num; GL_%t; VAR_hor; VAR_vert; GL_patt	LATP; STWH; WRKL_s; TSK_perf	24	RDSB
Jin, Xian, Jiang, Niu, Xu, & Yang, 2014 [81]	RADMS; PHCNV_hf; OTIV	driving simulator	TYPT	FIX_%t; FIX_num; FIX_max; PUP_size; GZ_andev; SACC; BL_rate; BL_dur	none	18	RDSB
Jin, Xian, Niu, & Bie, 2015 [82]	RADMS; PHCNV_hf; PHOTH; OTIV	driving simulator	TYPT	FIX_%t; FIX_num; FIX_max; PUP_size; GZ_andev; SACC; BL_rate; BL_dur	none	40	RDSB
Jones, Chapman, & Bailey, 2014 [83]	OTCOG	video clips	TYPT; HZRD	FIX_adur; VAR_hor; VAR_vert	OTHR; TSK_perf; SKC; HRT	36	RDSB
Kaber, Pankok, Corbett, Ma, Hummer, & Rasdorf, 2015 [84]	NAV_iv; ADSG	driving simulator	TYPT; ROAD; OTHR	GL_max; FIX_num	TSK_perf; LATP; SPE_stat	20	RDSB
Kaber, Liang, Zhang, Rogers, & Gangakhedkar, 2012 [85]	OTCOG	driving simulator	TYPT; TSKD	GL_num; GL_adur	STWH; HWTC; RET; TSK_t; WRKL_s	20	HMGL
Kettwich, Klinger, & Lemmer, 2008 [86]	ADSG	on-road	OTHR; TYPT	FIX_adur; FIX_tot; FIX_num; OTHR	none	16	RDSB
Kim & Yang, 2018 [87]	OTVIS; OTCOG; NAV_ptb; NAV_iv; PHCNV_hf	driving simulator	TYPT; MODT	GL_%t; GL_patt; SACC	STWH; HRT; LATP	11	RDSB
Knapper, Hagenzieker, & Brookhuis, 2015 [88]	PHTXT; PHCNV_h; NAV	driving simulator	TYPT	GL_%t; GL_num; GL_adur; GL_max	SPE_stat; LATP; WRKL_s; TSK_perf	20	RDSB
Kosaka, Koyama, & Nishitani, 2005 [89]	OTCOG	driving simulator	ROAD; TYPT	GL_num	INC; HRT	19	HMGL
Kountouriotis, Spyridakos, Carsten, & Merat, 2016 [90]	OTVIS; OTCOG	driving simulator	ROAD; TYPT	VAR_hor	TSK_perf; SPE_stat; STWH; LATP	12	RDSB
Krause, Angerer, & Bengler, 2015 [91]	RADMS; PMP; PHOTH	driving simulator	TYPT; MODT	GL_tot; GL_adur; GL_num	LATP; HWTC; TSK_t	24	HMGL
Kujala, Silvennoinen, & Lasch, 2013 [92]	NAV_iv	driving simulator	TSKD; MODT	GL_max; GL_over; GL_tot; GL_num; GL_adur	SPE_stat; SPE_ch; INC; LATP; STWH; WRKL_s	16	HMGL
Kun, Palinko, Medenica, & Heeman, 2013 [93]	OTCOG	driving simulator	TSKD; MODT; ROAD	PUP_size	none	8	RDSB
Lamble, Kauranen, Laakso, & Summala, 1999 [33]	PHDIA_h; OTCOG	on-road	OTPAR; TYPT	GL_adur	HWTC; RET; LATP; TSK_perf	19	RDSB
Lee, Roberts, Hoffman, & Angell, 2012 [94]	PMP	driving simulator	MODT; TSKD; ROAD	GL_num; GL_adur	LATP; SPE_stat; TSK_perf; TSK_t	50	RDSB
Lee, Yoon, & Shin, 2015 [95]	IVIS	driving simulator	MODT	FIX_tot; FIX_adur; FIX_num	SPE_ch; TSK_t; LATP	15	no information
Lee, Black, Lacherez, & Wood, 2016 [96]	OTCOG; OTVIS	video clips	AGE; TYPT	FIX_num; FIX_adur; SACC; VAR_hor; VAR_vert; TTFF; FIX_tot	RET; TSK_perf	40	RDSB
Lehtonen, Lappi, & Summala, 2012 [97]	OTCOG	on-road	ROAD; TYPT	VAR_hor; GL_patt	SPE_stat; TSK_perf	10	RDSB
Lehtonen, Lappi, Koskiahde, Mansikka, Hietamäki, & Summala, 2018 [98]	OTVIS; OTIV; OTTE	on-road	LOCOV; LOCIV; TYPT	FIX_num; FIX_adur	STWH; SPE_stat	14	RDSB
Lemercier, Pêcher, Berthié, Valéry, Vidal, Paubel, Cour, Fort, Galéra, Gabaude, Lagarde, & Maury, 2014 [99]	OTCOG	driving simulator	TYPT; OTHR	PUP_size; VAR_hor	SPE_stat; LATP; WRKL_s; TSK_perf	20	RDSB
Li, Zhu, Zhang, Wang, He, & Qu, 2018 [100]	NAV_ptb; PHOTH	driving simulator	MODT	GL_patt; VAR_hor; VAR_vert; GL_num; GL_adur	STWH; SPE_ch	14	RDSB
Li, Markkula, Li, & Merat, 2018 [101]	OTCOG	driving simulator	TSKD; ROAD	VAR_hor	LATP; STWH; SKC	27	HMGL
Li, Vaezipour, Rakotonirainy, & Demmel, 2019 [102]	IVIS	driving simulator	TRCON; ROAD; MODT	SACC; GL_tot; GL_adur; GL_num	none	36	RDSB
Li, Merat, Zheng, Markkula, Li, & Wang, 2018 [103]	OTCOG	driving simulator	TSKD	VAR_hor	LATP; STWH; TSK_perf	32	HMGL
Liang & Lee, 2010 [104]	OTCOG; OTVIS	driving simulator	TYPT	GL_adur; GL_num; BL_rate; VAR_hor; VAR_vert; SACC	STWH; LATP; RET; HWTC	16	RDSB
Libby, Chaparro, & He, 2013 [105]	PHCNV_h; PHTXT	driving simulator	TYPT	FIX_num; TTFF; OTHR	SPE_stat	20	no information
Ma, Xu, & Du, 2016 [106]	IVIS	driving simulator	MODT	FIX_tot; FIX_num; GL_patt	TSK_perf; INC; RET; TSK_t	15	HMGL
Mackun & Zukowska, 2018 [107]	ADSG	on-road	ROAD; AGE; TIME	FIX_tot; GL_patt	none	58	HMGL
Madleňák & Hudák, 2016 [108]	ADSG	on-road	LOCOV; TYPT	GL_num; FIX_adur; FIX_tot; FIX_over	none	3	HMGL
McGough & Ma, 2015 [109]	NAV_ptb; PHCNV_h	driving simulator	LOCIV; TYPT	FIX_%t; PUP_size	LATP; SPE_ch	20	HMGL
Metz, Schömig, & Krüger, 2011 [110]	NAV_iv; OTCOG	driving simulator	HZRD; TYPT	GZ_andev; FIX_adur; GL_%t	TSK_perf; INC	40	RDSB
Nabatilan, Aghazadeh, Nimbarte, Harvey, & Chowdhury, 2012 [111]	PHCNV_h; PHDIA_h	driving simulator	TRCON; DREXP; TYPT	FIX_%	INC; WRKL_s	38	HMGL
Najar & Sanjram, 2018 [112]	OTCOG	on-road	LOCOV; TSKD	FIX_adur; FIX_num; GL_num	LATP; SPE_stat; INC	30	HMGL
Niezgoda, Tarnowski, Kruszewski, & Kamiński, 2015 [113]	OTCOG	driving simulator	TYPT	BL_rate; PUP_size; FIX_adur; VAR_hor; VAR_vert	WRKL_s; TSK_perf; SPE_stat; STWH	46	HMGL
Oh, Ko, & Ji, 2016 [114]	NAV_iv; IVPAR	driving simulator	AGE; TSKD; MODT	GL_adur	RET; TSK_perf	25	HMGL
Ouimet, Pradhan, Simons-Morton, Divekar, Mehranian, & Fisher, 2013 [115]	PASS	driving simulator	OTPAR; OTHR; TYPT	VAR_hor; GL_num	INC; HWTC; OTHR	36	HMGL
Peng & Boyle, 2015 [116]	IVIS	driving simulator	TRCON; OTPAR; TSKD; TYPT	GL_max; GL_%t; GL_over	none	28	RDSB
Perez, 2012 [117]	IVIS; RADMS	on-road	TYPT	GL_num; GL_adur; GL_tot; GL_patt; GL_%; GL_%t	RET; TSK_perf	17	RDSB
Perlman, Samost, Domel, Mehler, Dobres, & Reimer, 2019 [118]	OTCOG; PHDIA_h	driving simulator	MODT	GL_num; GL_adur; GL_over; GL_tot; GL_%t; GL_max	TSK_perf; TSK_t; HRT; SKC; WRKL_s; RET; LATP; SPE_stat; STWH	36	RDSB
Pradhan, Li, Bingham, Simons-Morton, Ouimet, & Shope, 2014 [119]	PASS	driving simulator	OTHR; OTPAR	GL_tot; VAR_hor; VAR_vert	none	58	RDSB
Qin, Cui, Wang, Jia, & Hui, 2018 [120]	ADSG	driving simulator	TYPT	GL_tot; FIX_num; FIX_adur	RET; LATP	only the abstract was found	no information
Rahimi, Briggs, & Thom, 1990 [121]	ADSG; OTTE	on-road	TYPT; TRCON	GL_num	OTHR	1	HMGL
Recarte & Nunes, 2003 [30]	OTCOG	on-road	TYPT; MODT	PUP_size; VAR_hor; VAR_vert; GL_%; GZ_andev	WRKL_s; TSK_perf; RET	12	HMGL
Recarte & Nunes, 2000 [31]	OTCOG; OTVIS	on-road	ROAD; TYPT; TRCON	PUP_size; FIX_adur; GL_patt; VAR_hor; SACC; GL_num	SPE_stat	12	RDSB
Reimer, Mehler, & Donmez, 2014 [122]	PHDIA_h	driving simulator	MODT; GEND; OTPAR	GL_tot; GL_%t; GL_over	TSK_t; LATP; TSK_perf; SPE_stat; WRKL_s	36	RDSB
Reimer, Mehler, Wang, & Coughlin, 2012 [123]	OTCOG	on-road	AGE; TYPT	GL_patt; VAR_hor; VAR_vert	TSK_perf; RET; SPE_stat; STWH; SPE_ch	108	RDSB
Reimer, 2009 [124]	OTCOG	on-road	TSKD	GL_patt; VAR_hor; VAR_vert	LATP; SPE_stat; TSK_perf	21	RDSB
Reyes & Lee, 2008 [125]	IVIS; OTCOG	driving simulator	TSKD; HZRD	FIX_adur; GL_patt; SACC; GZ_andev	RET; TSK_perf; OTHR	12	RDSB
Sall, Wright, & Boot, 2014 [126]	OTTE	video clips	HZRD; TYPT	TTFF; SACC; OTHR	none	15	RDSB
Savage, Potter, & Tatler, 2013 [127]	OTCOG	video clips	TYPT	FIX_adur; SACC; VAR_hor; VAR_vert; BL_rate; BL_dur	RET; OTHR	17	RDSB
Schömig & Metz, 2013 [128]	OTCOG; OTVIS	driving simulator	HZRD; ROAD; OTPAR	GL_adur; GL_patt; GL_%; GL_tot	TSK_perf; TSK_t; OTHR	15	RDSB
Schömig, Metz, & Krüger, 2011 [129]	OTVIS	driving simulator	TYPT; HZRD	FIX_%t; VAR_hor; VAR_vert	TSK_perf; TSK_t; SPE_stat; LATP; OTHR; INC	24	RDSB
Smiley, Persaud, Bahar, Mollett, Lyon, Smahel, & Kelman, 2005 [130]	ADSG	on-road	LOCOV; TYPT	OTHR; GL_adur; GL_patt	HWTC	16	HMGL
Smiley, Smahel, & Eizenman, 2004 [131]	ADSG	on-road	ROAD; TYPT; HZRD	GL_num; GL_patt; GL_adur; GZ_andev; OTHR	HWTC	16	HMGL
Sodhi, Reimer, Cohen, Vastenburg, Kaars & Kirschenbaum, 2002 [22]	RADMS; OTTE; IVPAR; PHDIA_h; PHDIA_hf	on-road	TYPT	VAR_hor; VAR_vert; GL_adur	none	24	HMGL
Sodhi, Reimer & Llamazares, 2002 [23]	RADMS; OTTE; IVPAR; PHDIA_h; PHDIA_hf	on-road	TYPT	GL_patt; GL_tot; GL_max; GL_num; GL_adur; SACC	TSK_t	5	HMGL
Strayer, Drews & Johnston, 2003 [27]	PHCNV_h	driving simulator	LOCOV; TYPT	FIX_tot; OTHR	TSK_perf; OTHR	20	RDSB
Strayer, Cooper & Drews, 2004 [132]	PHCNV_hf; PHDIA_hf	driving simulator	LOCOV; HZRD; TYPT	OTHR; FIX_tot; GL_patt	TSK_perf; OTHR	32	RDSB
Tangmanee & Teeravarunyou, 2012 [133]	NAV_iv	driving simulator	MODT; GEND; DREXP	FIX_adur; FIX_tot; FIX_num	RET	5	HMGL
Tijerina, Parmer & Goodman, 1999 [134]	RADMS; NAV_ptb; NAV_iv; PHDIA_h	on-road	MODT; TYPT	GL_adur; GL_num	TSK_t; LATP	16	RDSB
Tivesten, & Dozza, 2014 [135]	PHDIA_h; PHTXT	on-road	TRCON; TYPT; ROAD	GL_%t; GL_max; GL_over; GL_num; GL_tot	SPE_stat	198 overall	RDSB
Topolšek, Areh, & Cvahte, 2016 [136]	ADSG; OTTE	on-road	AGE; LOCOV	GL_patt	OTHR	17	HMGL
Victor, Harbluk, & Engström, 2005 [32]	OTVIS; OTCOG	both on-road and driving simulator	TSKD; ROAD	GL_adur; OTHR; GL_over; GL_num; GL_tot; GL_patt; VAR_hor; VAR_vert	none	24 on-road, 95 simulator	RDSB
Wang, Bao, Du, Ye, & Sayer, 2017 [137]	PHCNV_h; PHTXT	on-road	TIME; TYPT	SACC; GL_num; GL_adur	none	19	RDSB; HMGL
Wang, Mehler, Reimer, Lammers, D’Ambrosio, & Coughlin, 2010 [138]	IVIS; NAV_iv	both on-road and driving simulator	ROAD; MODT	GL_%t; GL_num; GL_tot; GL_over	LATP; RET; TSK_t; SPE_stat	58	RDSB
White & Caird, 2010 [139]	PASS	driving simulator	GEND; HZRD; TYPT	FIX_num	TSK_perf; INC; SPE_stat; OTHR	40	HMGL
Wikman, Nieminen, & Summala, 1998 [36]	RADMS; OTMAN; PHDIA_h	on-road	DREXP; GEND; TYPT; ROAD	GL_patt; GL_adur; GL_over; GL_num	LATP; TSK_t	47	RDSB
Wong & Huang, 2013 [140]	OTTE	on-road	ROAD; TRCON; TYPT	GL_patt; GL_adur; GL_tot; OTHR	none	no data, used the 100-car naturalistic glance data	no information
Wood, Hartley, Furley, & Wilson, 2016 [141]	OTCOG	video clips	OTPAR; TYPT	TTFF; FIX_adur; PUP_size	OTHR; TSK_perf	24	HMGL
Wright, Vitale, Boot & Charness, 2015 [142]	OTTE	video clips	TIME; TSKD; AGE	SACC; TTFF; OTHR	none	34	RDSB
Wu, Zhu, Lu, & Zhu, 2016 [143]	OTCOG; PHCNV_h	driving simulator	TYPT	GL_patt; VAR_hor; VAR_vert; FIX_tot	WRKL_s; RET	25	HMGL
Xian & Jin, 2015 [144]	IVIS	driving simulator	TRCON; TSKD	FIX_%t; FIX_num; FIX_max; FIX_adur; GZ_andev; PUP_size; SACC	STWH; SPE_stat; LATP; TSK_t	18	RDSB
Yamani, Horrey, Liang, & Fisher, 2015 [145]	IVPAR; OTMAN; RADMS	driving simulator	OTPAR; TYPT; TSKD	GL_adur; GL_patt	none	40	HMGL
Yang, McDonald, & Zheng, 2012 [146]	OTVIS	on-road	TSKD	GL_%t; BL_rate; SACC	RET; TSK_perf; SPE_stat; LATP; STWH; WRKL_s	34	RDSB
Yang, Reimer, Mehler, Wong, & McDonald, 2012 [147]	OTVIS; OTCOG	on-road	TYPT; TSKD	GZ_andev	WRKL_s; SPE_stat; LATP; HWTC	34	RDSB
Yang, Wong, & McDonald, 2015 [148]	OTCOG	on-road	TSKD; GEND	GZ_andev; BL_rate; GL_%t	SPE_stat; HWTC; LATP; TSK_perf	34	RDSB
Yoshizawa, & Iwasaki, 2017 [149]	OTTE	video clips	HZRD; TYPT	SACC	RET	4	RDSB
Young, Lenné, Salmon, & Stanton, 2018 [150]	PHTXT	driving simulator	HZRD; TYPT	GL_%t; GL_adur; GL_num	RET; LATP; SPE_stat; TSK_perf	28	RDSB
Young, Mahfoud, Stanton, Salmon, Jenkins, & Walker, 2009 [151]	ADSG	driving simulator	ROAD; TYPT	FIX_num; GL_adur	LATP; HWTC; WRKL_s; TSK_perf	48	HMGL
Young, Mitsopoulos-Rubens, Rudin-Brown, & Lenné, 2012 [152]	PMP	driving simulator	TSKD; HZRD	GL_%t; GL_num; GL_adur; GL_over	SPE_stat; LATP; HWTC; TSK_t; OTHR	20	RDSB
Zahabi, & Kaber, 2018 [153]	IVIS	driving simulator	TYPT; MODT; HZRD	GL_num; GL_max	LATP; SPE_stat; RET; TSK_perf; WRKL_s; OTHR	20	RDSB
Zahabi, Machado, Lau, Deng, Pankok, Hummer, Rasdorf, & Kaber, 2017 [154]	ADSG	driving simulator	AGE; TYPT; OTHR	FIX_num; GL_max	TSK_perf; SPE_stat; LATP; SPE_ch	60	RDSB
Zahabi, Pankok, Kaber, Machado, Lau, Hummer, & Rasdorf, 2017 [155]	ADSG	driving simulator	AGE; ROAD; TYPT; OTHR	GL_max	TSK_perf	120	RDSB
Zahabi, Machado, Pankok, Lau, Liao, Hummer, Rasdorf, & Kaber, 2017 [156]	ADSG	driving simulator	TYPT; AGE; OTHR	FIX_num; GL_max	TSK_perf; SPE_stat; LATP	60	RDSB
Zhang, Reimer, Mehler, Dobres, Pala, Angell, & Ifushi, 2013 [157]	NAV_ptb; PHOTH	driving simulator	TYPT	GL_tot; GL_num; GL_over	SPE_stat; TSK_t; WRKL_s	24	RDSB
Zhang, & Kontou, 2014 [158]	OTTE	on-road	TIME; ROAD; TRCON; TYPT	GL_patt	SPE_stat	35	RDSB
Zhang, Harris, Rogers, Kaber, Hummer, Rasdorf, & Hu, 2013 [159]	ADSG	driving simulator	TYPT; OTHR	GL_max; GL_%	TSK_perf; TSK_t; SPE_stat; LATP; INC; RET	24	HMGL
Zhang, Kaber, Rogers, Liang, & Gangakhedkar, 2014 [160]	OTVIS; OTCOG; OTMAN	driving simulator	TYPT; OTHR	GL_adur; GL_%	TSK_perf; STWH; SPE_stat; TSK_t	20	HMGL
Zhang, Smith, & Witt, 2006 [161]	OTVIS; OTCOG	driving simulator	ROAD; TSKD; LOCIV; TYPT	GL_num; GL_adur; GL_tot; GL_%t; GZ_andev; OTHR	RET; WRKL_s; TSK_perf; LATP; STWH	14	RDSB
Zhang, Zhang, Liu, & Guo, 2017 [162]	ADSG	on-road	TIME; TYPT	GL_patt; PUP_size; BL_rate	LATP	no information	no information
Zhou & Itoh, 2015 [163]	OTCOG; IVIS	driving simulator	MODT; TYPT	GL_num	HWTC	18	RDSB
Zhou, Itoh, & Inagaki, 2008 [164]	OTCOG	driving simulator	TYPT	GL_num; GL_patt	STWH; SPE_stat; SPE_ch; WRKL_s	8	RDSB
Zhou, Itoh, & Inagaki, 2009 [165]	OTCOG	driving simulator	TYPT	FIX_num	HWTC; WRKL_s; LATP	17	RDSB
